# Recent Reversal of the Upper-Tropospheric Temperature Trend and its Role in Intensifying the East Asian Summer Monsoon

**DOI:** 10.1038/srep11847

**Published:** 2015-07-02

**Authors:** Siyao Zhao, Jian Li, Rucong Yu, Haoming Chen

**Affiliations:** 1 State Key Laboratory of Severe Weather, Chinese Academy of Meteorological Sciences, No. 46 Zhong-Guan-Cun South Street, Beijing, 100081, China

## Abstract

At the beginning of the 21st century, the July and August (JA) mean upper-tropospheric temperature over East Asia shows a significant increasing trend, contrary to the decreasing trend in the late 1970 s. The largest warming center is over northern China (between 30°N–45°N and 85°E–120°E) around 300 hPa. Together with the temperature rising, the geo-potential height rises above the warming center and drops below, which connects closely to a correspondingly significant decadal shift of the general circulation over East Asia. In the upper-level of the troposphere, an anomalous anti-cyclone dominates, and the 200–hPa westerly jet strengthens due to the increasing pole-ward geo-potential height gradient. In the lower-troposphere, the anomalous southerly wind increases around Yangtze River Valley and the East Asian summer monsoon intensifies. The integrated circulation changes seriously impact summer precipitation over East Asia. The so-called “southern flood and northern drought” (SFND) pattern since the 1970 s over eastern China has changed. As the cooling center in the 1970 s moves southward, the dry belt moves southward as well. A wet belt dominates the Huaihe River Valley after the temperature trend reversal at 2005 while southern China experiences a dry condition.

East Asia, with its high population density and rapid economic development, is greatly sensitive to climate change. Severe climate change may have disastrous impact on East Asia. For instance, droughts and floods have caused enormous economic losses and casualties in China. Under the background of global warming, the East Asian climate experiences a distinct inter-decadal shift in the late 1970 s. Summer precipitation manifests as a “southern flood and northern drought” (SFND) anomalous pattern in eastern China. The precipitation increases over the southern part of central-eastern China (i.e. the middle and lower reaches of the Yangtze River), and decreases over the north (the lower reaches of the Yellow River)^1^,^2^,^3^. Correspondingly, the general circulation over East Asia also changes. The East Asian subtropical westerly jet moves southward^4^, and the East Asian summer monsoon weakens^5^,^6^.

The mechanisms of East Asian inter-decadal climate changes in the 1970 s were extensively discussed in the last decades. While some studies emphasized the influences of anthropogenic factors like increasing emission of black carbon^7^ and sulfate aerosols^8^, numerous studies attributed the influences to the natural factors, such as the abnormal thermodynamic state of the Tibetan Plateau, especially aberrant winter-spring snow cover^9^, the Pacific Decadal Oscillation (PDO)^10^ and ENSO^11^,^12^. Yu, *et al.*^13^ found a significant inter-decadal cooling of the upper-tropospheric temperature, and raised the possibility that the integrated East Asian monsoon circulation and rainfall changes could be linked to the upper-tropospheric cooling. This cooling anomaly corresponds to the generation of an anomalous cyclone above the cooling center and anti-cyclone below. Accordingly, the upper-level subtropical westerly jet shifts southward resulting in stronger convergent and ascending flows over the Yangtze River Valley^14^, while lower-level anomalous northerly wind weakens the north progress of the monsoon wind and brings less water vapor to northern China^15^. Thus, the rain-belt hovers around Yangtze River Valley and leads to the SFND precipitation anomalous pattern. Based on composite analyses Sun, *et al.*^16^ verified that a similar coherent three-dimensional circulation structure forms before heavy summer rainfall in central northern China, which is characterized by a remarkable upper-tropospheric warming and upper/lower-level circulation changes. In addition, the mechanism linking between an upper tropospheric cooling and a decadal decrease of late spring rainfall in southeast China has also been testified by model experiments^17^. Research shows that the potential causes of the upper-tropospheric temperature decrease may include the stratospheric cooling^13^ and the snow depth change over Tibetan Plateau^18^.

The above mentioned coherent changes of monsoon rainfall and its associated large-scale circulation in the 1970 s imply that the inter-decadal climate variation in East Asia could mostly arises from the regional response to the global climate change^19^. Since the 2000 s, the rate of the global warming has slowed down (i.e. global warming hiatus^20^,^21^). Evidences also demonstrated that the climate over East Asia has shown different or even opposite changes compared to the temperature rapid rising (global warming) stage^22^,^23^. Observational analyses in recent years revealed that the SFND pattern has changed. Summer flooding events have increased in the Huaihe River valley^24^ and the monsoon rain belt has tended to move northward in eastern China^23^. The East Asian summer monsoon has also strengthened lately^22^. Wetter condition has prevailed in Huaihe River Valley, causing more frequent rainfall, mudslides, urban floods and other meteorological disasters. Since these may have a severe impact on socio-economic development, it is crucial to focus on these climate reversals taking place in East Asia. The main motivation of this study is to investigate the upper-tropospheric temperature trend reversal and its relationship with surface climate changes in the new global warming hiatus stage.

## Data and Methods

We utilized monthly temperature and circulation reanalysis data from the United States National Centers for Environmental Prediction/National Center for Atmospheric Research (NCEP/NCAR)^25^. The dataset is 2.5° × 2.5° horizontally and contains 17 vertical levels. The precipitation data came from daily rain gauge records from 384 stations in central east China during 1951–2013 which was archived and quality controlled by the National Meteorological Information Center of the China Meteorological Administration.

Statistic test methods were applied to verify the statistical significance of the inter-decadal changes. The autocorrelation in the time series, which reduces the data’s independent sample number^26^,^27^,^28^, was accounted for through the computation of the effective sample size (ESS), using the method outlined in Trenberth^26^ and Bretherton, *et al.*^27^.

## Results

**Figure 1:**
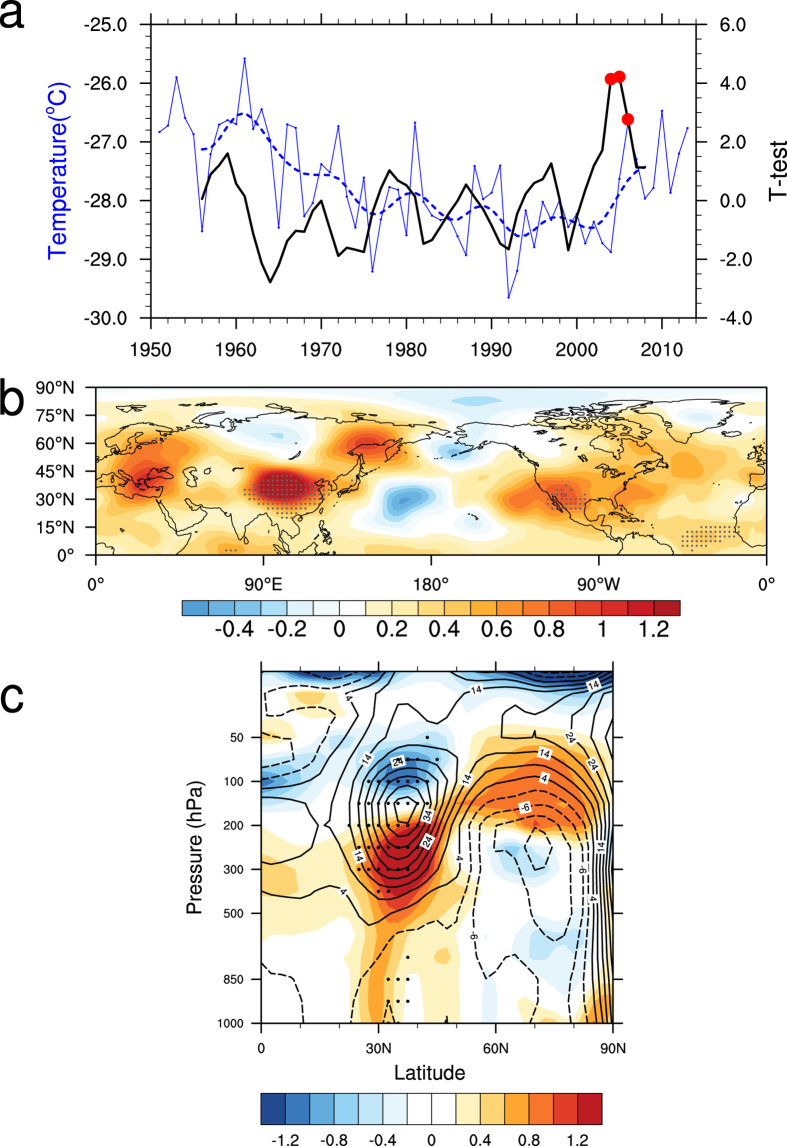
Inter-decadal change of the JA mean upper-tropospheric temperature. **(a)** Time series of the JA mean (blue solid line; °C) and 10 year low-pass filter (blue dashed line; °C) of the upper-tropospheric (500–200 hPa) temperature over East Asia (30°N–45°N, 85°E–120°E) from 1951 to 2013. Black solid line is the result of an 11 year moving *t*-test, the red dots are statistically significant at the 99% confidence level. **(b)** The inter-decadal change (2005–2013 minus 1994–2004) of the JA mean upper-tropospheric (500–200 hPa) temperature (°C). Black dots are the regions statistically significant at the 99% confidence level (using Student’s *t*-test). **(c)** Latitude-height cross section of the JA mean temperature (shading; °C) and geo-potential height (black contours; gpm) change (2005–2013 minus 1994–2004) averaged between 85°E–120°E. The ordinate is on a log-pressure (hPa) scale. Black dots show geo-potential height change statistically significant at the 95% confidence level (using Student’s *t*-test). The map in the figure were created by Z.S. using The NCAR Command Language^34^(http://www.ncl.ucar.edu/).

At the beginning of the 21^st^ century, the upper-tropospheric temperature over East Asia has experienced a significant trend reversal. Figure 1a shows the July and August (JA) mean upper-tropospheric (500–200 hPa) temperature over East Asia (30°N–45°N and 85°E–120°E) from 1951 to 2013 (blue solid line). The decrease in temperature since the 1950 s has transformed into a significant warming trend in the past decade. Similar results were derived with ERA-Interim reanalysis data (not shown). According to the 11 year moving *t*-test results (Fig. 1a black solid line), the first point which is statistically significant at the 99% confidence level is 2004. And considering the moving *t*-test results and the original time series, the inter-decadal change of the upper-tropospheric temperature over East Asia took place between 2004/2005. The mean temperature in the latest decade is −27.3 °C and almost recovers to its early 1970 s level after the cooling trend. A 10 year low-pass filter was applied to the original upper-tropospheric temperature time series (Fig. 1a blue dashed line). By filtering the high frequency oscillation, the trend reversal is even more evident. With the ordinary periodic oscillation, the upper-tropospheric temperature shows a general decreasing trend before the beginning of the 21^st^ century, and an increasing trend after.

To further analyze the spatial distribution of the upper-tropospheric temperature change, we calculated the inter-decadal change by the difference between 2005–2013 and 1994–2004. The warming trend spreads over most areas of the Northern Hemisphere (Fig. 1b) with the strongest warming center over northern China and Mongolia. Regions with temperature rising over 1.0 °C cover most areas between 32°N–50°N and 90°E–125°E. The warming center is located in the south of the cooling center found by Yu, *et al.*^13^, who defined the cooling center as the upper-tropospheric temperature difference between 1980–2001 and 1958–1979.

**Figure 2:**
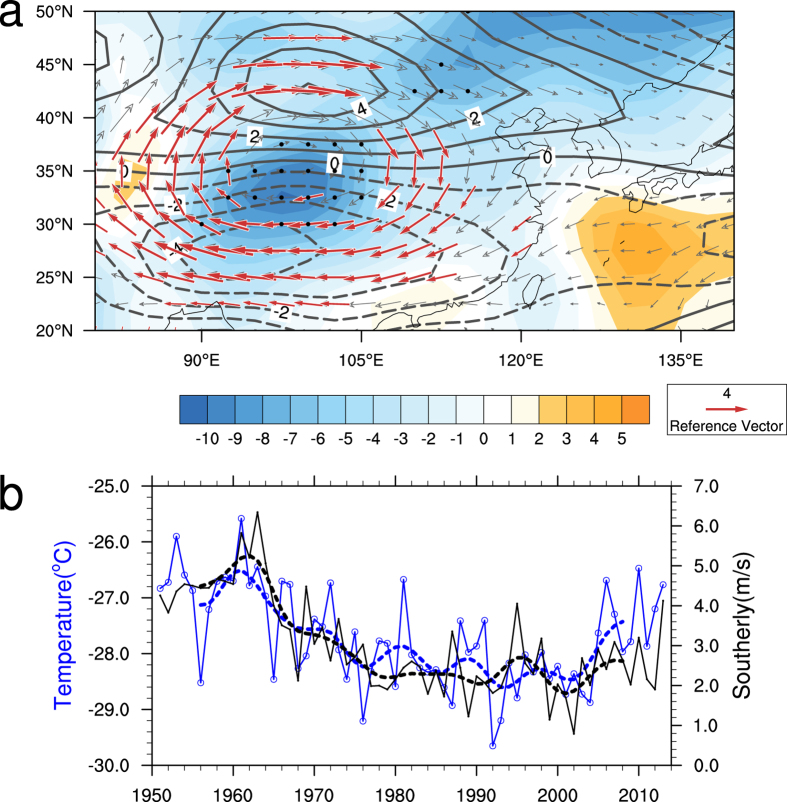
Large-Scale circulation change associated with the upper-tropospheric temperature change. **(a)** Inter-decadal changes (2005–2013 minus 1994–2004) of the JA mean 200–hPa westerly (contours; m/s), 200–hPa wind (vectors; m/s) and 1000–850 hPa geo-potential height (shading; gpm). Black dots show geo-potential height change and red vectors show 200–hPa wind change that are statistically significant at the 95% confidence level (using Student’s *t*-test). **(b)**Time series of the upper-tropospheric (500–200 hPa) temperature averaged over 30°N–45°N and 85°E–120°E (blue solid line; °C) and the lower-tropospheric (925–850 hPa) southerly wind averaged over 25°N–35°N and 110°E–125°E (black solid line; m/s) from 1951 to 2013. Dashed lines are 10 year low-pass filter results for the upper-tropospheric temperature (blue) and southerly (black). The map in the figure were created by Z.S. using The NCAR Command Language^34^(http://www.ncl.ucar.edu/).

In the vertical direction, the warming extends from the surface to 200 hPa over mid-latitude East Asia (Fig. 1c). The largest temperature increasing center over East Asia appears between 500 hPa to 200 hPa, with a maximum at 300 hPa. Above the warming anomaly, a cooling center locates in the stratosphere. The temperature rises in the upper-troposphere to the south of 45°N which corresponds with an upward bulging of the upper-tropospheric pressure level. According to geostrophic adjustment theory, the wind field adjusts to pressure in large-scale motions. Meanwhile, combining theoretical derivation and model experiment, studies have demonstrated the adjustment process of wind field to temperature field^29^. So the upper-level temperature change has greatly influences on the circulation change. Based on hydrostatic and geostrophic equilibriums, the warming anomaly corresponds to the generation of an anti-cyclonic anomaly in the upper-level (vectors in Fig. 2a). Temperature and geo-potential height change in the regions north of 45°N is in contrary circumstances compared to the south. The pole-ward geo-potential height gradient increases and results in the enhancement of the 200–hPa westerly jet (contours in Fig. 2a) to the north of the warming center through the geostrophic balance.

The lower-level monsoon circulation also changes according to the upper-tropospheric warming-induced mass change. Right below the warming center, geo-potential height decreases (shading in Fig. 2a) and the anomalous southerly wind is enhanced on the southeast side of the decrease of geo-potential height over eastern China. The southerly wind over the East Asian monsoon region is widely used to represent the intensity of the East Asian summer monsoon^11^,^30^. The increasing southerly over eastern China signifies the intensification of the East Asian summer monsoon. For further understanding of the East Asian summer monsoon change associated with the warming trend, the relationship between the upper-tropospheric temperature and the lower-level (925–850 hPa) southerly wind over East Asia (averaged between 25°N–35°N and 110°E–125°E) was investigated (Fig. 2b). The lower-level meridional wind (black solid line) presents similar trend to the upper-tropospheric temperature (blue solid line) during the whole investigated period. The decreasing trend extends from the 1960 s to the end of the 1990 s and then reverses to an increasing trend at the beginning of the 21^st^ century. The regional mean southerly wind during 2005–2013 is 0.45 m/s higher than it was in 1994–2004. The 10-year low-pass filtered results of the southerly (black dashed line) and the upper-tropospheric temperature (blue dashed line) exhibit an even more similar trend. To further statistically confirm the connection between the change of upper-tropospheric temperature and the low-level southerly, the correlation coefficient was calculated. The correlation coefficient between the detrended time series reaches 0.48. As the two time series both have strong low-frequency component, their ESSs are much smaller than the sample size^26^,^27^. We calculated the ESS for the detrended temperature and southerly wind time series respectively. The ESS is 32 for the detrended upper-tropospheric temperature time series and 29 for the detrended lower-level southerly wind time series. And even if we used the smaller ESS which is 29, the correlation coefficient is statistically significant at 99% confidence level (P = 0.0084).

**Figure 3:**
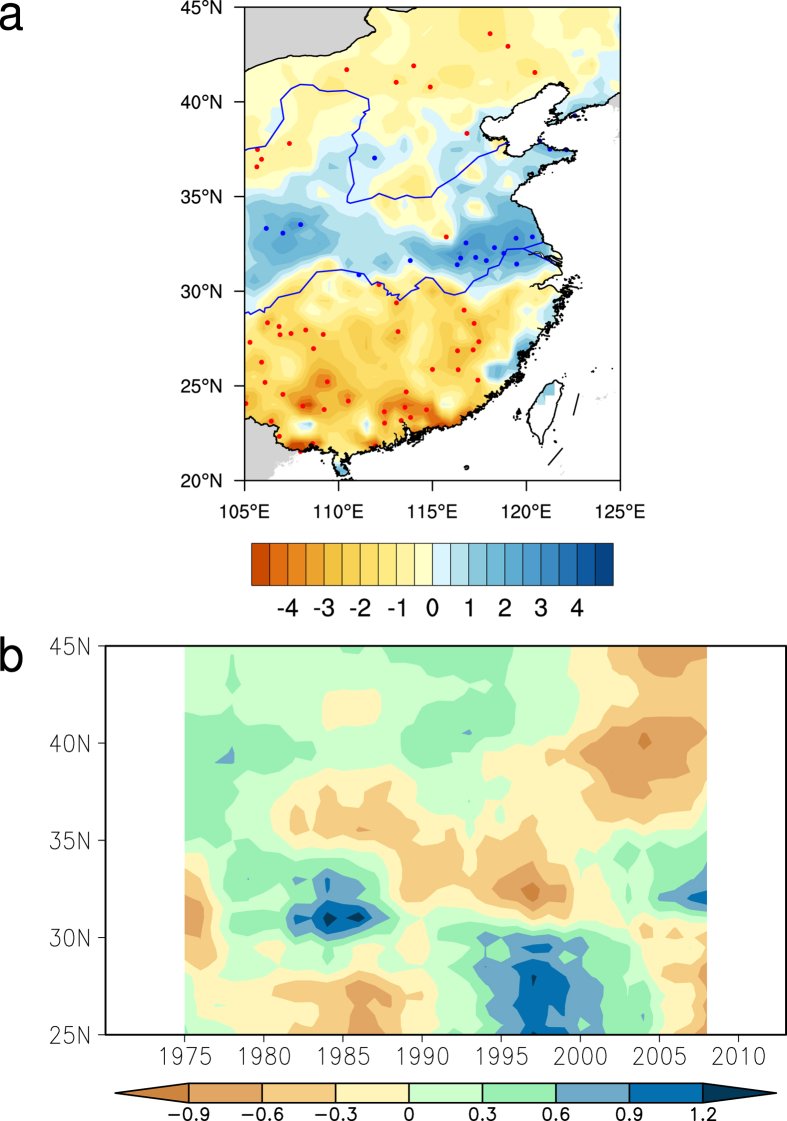
Precipitation change associated with the upper-tropospheric temperature change. **(a)** Inter–decadal change (2005–2013 minus 1994–2004) of the JA mean precipitation (mm/d) over eastern China. Red (blue) dots are the stations significant decrease (increase) at the 90% confidence level (using Student’s *t*-test). **(b)** 11 year smoothed precipitation anomalies (mm/d) averaged between 105°E–120°E. The map in the figure were created by Z.S. using The NCAR Command Language^34^ (http://www.ncl.ucar.edu/).

Corresponding to the monsoon circulation change, the precipitation over eastern China also changes. As mentioned before, the upper-level westerly jet strengthens in the north of the warming center and generates strong ascending flows over northern China. The East Asian summer monsoon strengthens and brings more water vapor across the Yangtze River. These circulation changes lead more rainfall to Huaihe River Valley (30°N–35°N) and less in southern China with a clear boundary along Yangtze River (30°N) (Fig. 3a). The region with significant increase of precipitation is centered along Huaihe River Valley (30°N–35°N) and the stations with significant decrease are widely distributed in the regions south of Yangtze River (20°N–31°N).

To further reveal the trend reversal of precipitation over eastern China, the temporal and spatial change of the precipitation anomalies are shown in a Hovmöller plot (Fig. 3b). At the end of the 1970 s, the precipitation shows negative anomalies in northern China and positive anomalies in Yangtze-Huaihe River Basin with a boundary around 35°N which indicates the notable SFND precipitation anomalous pattern. The entire pattern moves southward since then. The negative anomalies move to the Yangtze-Huaihe River Basin and positive anomalies dominate southern China. The boundary line between the negative and positive anomalies moves southward from 35°N in the late 1970 s to 31°N. After 2005, the pattern reverses as positive anomalies along Huaihe River and negative anomalies over regions south of the Yangtze River. The location of the SFND-liked precipitation pattern is not fixed. It moves as the upper-tropospheric temperature anomalous center moves (figure not shown). As the cooling center in the late 1970 s shifts southward, the whole pattern moves along. And when the cooling center reverses to a warming center, the pattern turns opposite from before. This indicates the strong connection between the upper-level temperature and monsoon precipitation.

## Conclusion

In this study, we thoroughly examined the reversal of the East Asian upper-tropospheric temperature, from the downtrend during the global surface temperature rapid increasing stage to the upward trend after the global warming hiatus. Characterized by the upstream middle and high latitudes upper-tropospheric temperature change, a favorable circulation structure covers East Asia and eventually affects summer rainfall. The linkage between the upper-tropospheric temperature and the East Asian summer monsoon, as well as monsoon rainfall, was discussed in this work. The geo-potential height changes correspondingly with the upper-tropospheric temperature. In the upper-level, the geo-potential height increases. By the hydrostatic and geostrophic equilibriums, the warming favors the generation of anti-cyclonic anomalies in the upper-troposphere which enhance the 200–hPa westerly jet. Lower-level geo-potential height decreases, indicating the East Asian summer monsoon has intensified. These circulation changes play a decisive role in the precipitation anomalous pattern in East Asia as more rainfall in the Huaihe River Valley and less in southern China.

According to previous studies, several factors may be connected with the upper-tropospheric temperature’s inter-decadal change. Yu, *et al.*^13^ suggested that the cooling trend in upper-troposphere in the 1970 s is linked with the cooling in stratosphere. Xin, *et al.*^18^ showed that the snow depth over Tibetan Plateau is in negative correlation with late spring tropospheric temperature change over East Asia. PDO is also one of the factors that proved to be closely linked with the East Asian inter-decadal climate change in 1970 s. The integrity structure of the SFND in 1970 s can be reproduced by historical SST forcing, especially the PDO pattern^31^,^32^. The positive phase of PDO may be linked to negative summer rainfall anomalies in northern China and the weakness of the East Asian summer monsoon^10^. However, other study has pointed out that the current status of PDO is affected largely by atmosphere but not the other way around^33^. Thus, whether there is a connection between the upper-tropospheric temperature and PDO remains elusive and needs follow up studies. Sensitivity experiments using a climate model may aid in understanding of the contribution and influence of these factors on upper-tropospheric temperature change and the corresponding inter-decadal variation of East Asian climate.

## Additional Information

**How to cite this article**: Zhao, S. *et al.* Recent Reversal of the Upper-Tropospheric Temperature Trend and its Role in Intensifying the East Asian Summer Monsoon. *Sci. Rep.* **5**, 11847; doi: 10.1038/srep11847 (2015).
